# The Relationship Between Colles’ Fractures and Leukocytosis in the Emergency Department

**DOI:** 10.7759/cureus.29611

**Published:** 2022-09-26

**Authors:** Min-Yi Hsieh, Hui-Ying Tsai, Yen-Cheng Lin, Chuin Hua Tang, Hung-Chun Chung

**Affiliations:** 1 Department of Emergency, Cardinal Tien Hospital, New Taipei, TWN; 2 Department of Education and Research, Cardinal Tien Hospital, New Taipei, TWN

**Keywords:** antibiotics, blood testing, emergency care, leukocytosis, colles’ fracture

## Abstract

In Taiwan, emergency physicians often perform wrist joint reduction and cast immobilization before orthopedic surgeons arrange for surgical management. Prophylactic antibiotics can decrease the risk of wound infection and have been routinely employed in orthopedic surgery. In Taiwan, emergency physicians also regularly perform blood investigations and administer prophylactic antibiotics to prevent infection if the patient exhibits leukocytosis. However, pain and pressure also cause leukocytosis, making it difficult to discern if the cause is infection or injury. Therefore, we explored the relationship between Colles’ fractures and leukocytosis to determine if antibiotic treatment is necessary for this type of injury.

## Introduction

Displaced distal radius fractures (i.e., Colles’ fractures) are the most common upper limb fractures in middle-aged and older individuals due to falling accidents [1]. Traditionally, forearm fractures were treated non-operatively with reduction and cast immobilization, which has become the gold standard for elderly patients [2]. However, hospital admission and surgical treatment are highly recommended for patients with displaced forearm fractures. Nonetheless, there remains no consensus on the best treatment method for elderly patients with distal radius fractures [3].

In Taiwan, emergency physicians often perform wrist joint reduction and cast immobilization for patients with a Colles’ fracture before the orthopedic surgeon arranges an operation. In addition, emergency physicians regularly perform blood investigations to rule out other clinical diseases before admission or as part of the preoperative preparations. The basic blood investigations include a complete blood count, differential blood count, and sequential multiple analysis, which are used to evaluate kidney and liver function.

Prophylactic antibiotics can decrease the risk of wound infection and have been routinely employed in orthopedic surgery for decades [4]. Prophylactic antibiotic treatment is mandatory in every operation involving an orthopedic implant [5]. Few studies have explored the relationship between bone fractures and leukocytosis [6]. Therefore, we analyzed the relationship between the white blood cell (WBC) count and Colles’ fractures to determine if treating leukocytosis, such as with antibiotics, is necessary before admission to the orthopedic ward.

## Materials and methods

Data collection

We retrospectively collected patient data related to distal radius fractures from the Cardinal Tien Hospital in New Taipei City, Taiwan, from 2016 to 2020. The patients were identified using the following International Classification of Diseases, Tenth Revision (ICD-10) diagnostic codes: S52.532A, S52.531A, S52.502A, and S52.501A; we identified 405 patients. Patients aged 20-95 years with a distal radius fracture (left or right) were included in the study. Fever is a process where normal body temperature is raised over homeostasis conditions. Fever is an important resource for infectious diseases [7]. Signs and symptoms of infection may vary depending on the location of the infection and the type of bacteria or virus that’s causing it. Some general symptoms of infection include: (1) fever, (2) feeling tired or fatigued, (3) swollen lymph nodes in the neck, armpits, or groin, (4) headache, and (5) nausea or vomiting. Patients with above infection signs and symptoms were excluded when we reviewed the patient’s chart records.

Patients with fever-related symptoms or unstable vital signs upon arrival to the emergency room, multiple bone or joint fractures (i.e., more than one bone or joint), open fractures, immune insufficiency, or those taking antibiotics were excluded in our study. Dengue is not considered endemic in Taiwan [8]. Dengue fever usually has following signs or symptoms: headache, muscle, bone or joint pain, nausea, vomiting, pain behind the eyes, swollen glands, and rash. Patients with above dengue fever symptoms were also excluded via review chart records.

Orthopedic ward pre-admission processes

First, bone fractures are identified by X-ray imaging. Then, after distal radius fracture (displaced or non displaced) is confirmed and the patient agrees to admission, they will receive a cast splint immobilization and undergo blood investigations. The basic blood investigations performed in the emergency room provide the orthopedic surgeon with more information regarding the patient’s current medical status, helping them plan for surgery. Moreover, the patients must tolerate pressure and pain when cast splints are applied over the wrist joint. Therefore, blood is regularly taken for testing after its application. Finally, the patient will absolutely be admitted regardless the laboratory data is normal or not, such as a normal-range WBC level and stable vital signs. If the laboratory data is not normal, such as poor renal function, emergency physicians will let the orthopedics patient combine care with nephrologist.

Statistical analyses

We evaluated the WBC count based on the distal radius fracture location by one-way analysis of variance. Furthermore, we analyzed the relationships between age and sex and the WBC count by correlation analyses with unpaired t-tests.

## Results

**Figure 1:**
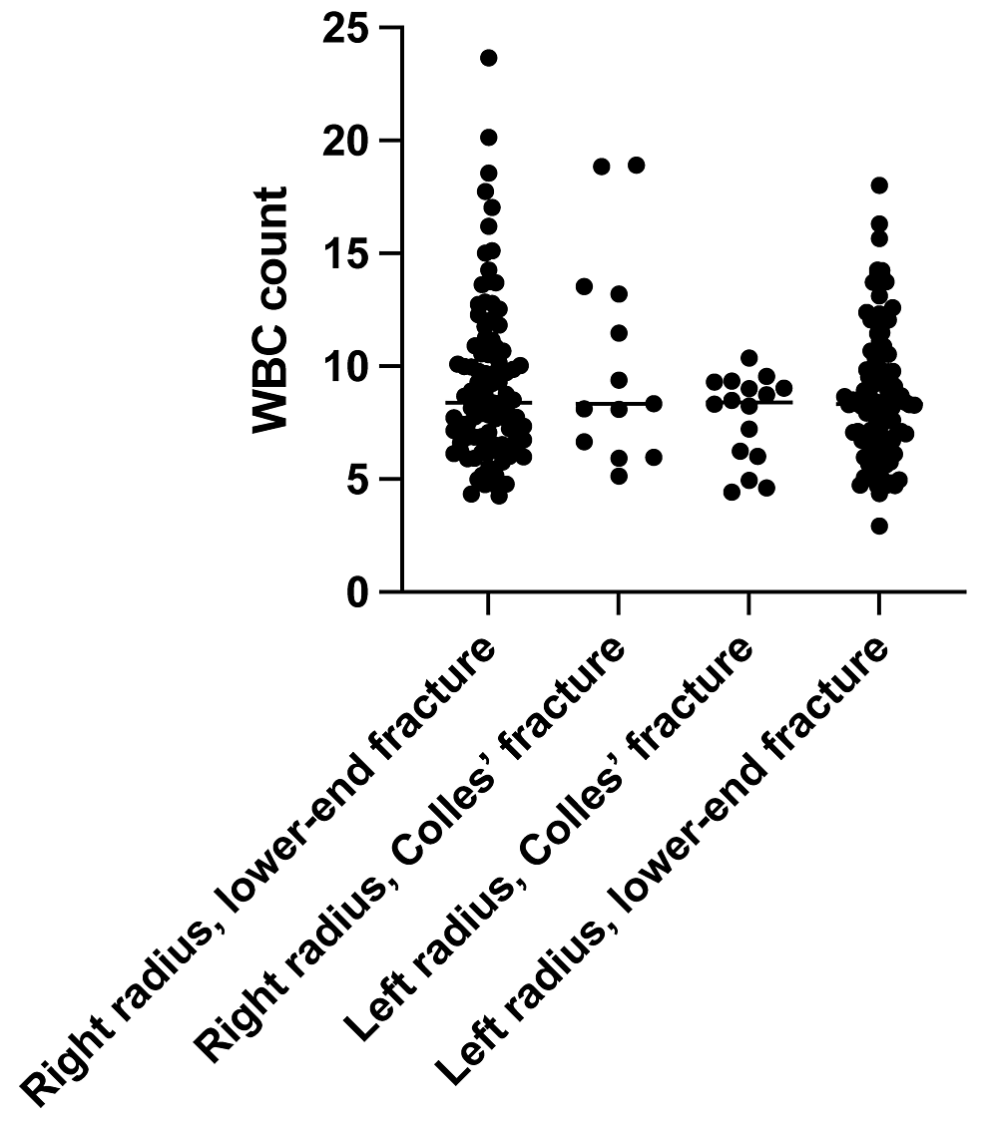
The white blood cell (WBC) count based on the distal radius fracture location

**Figure 2:**
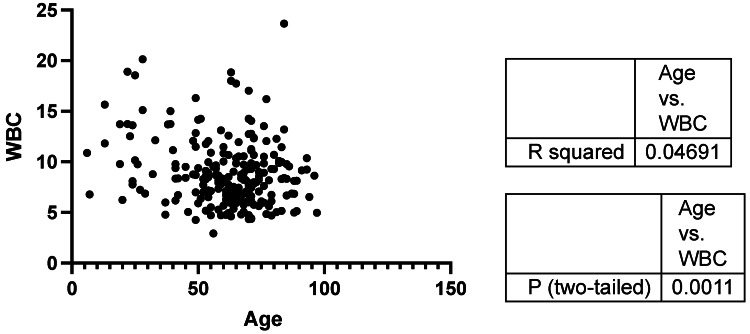
The relationship between age and the white blood cell (WBC) count

**Figure 3:**
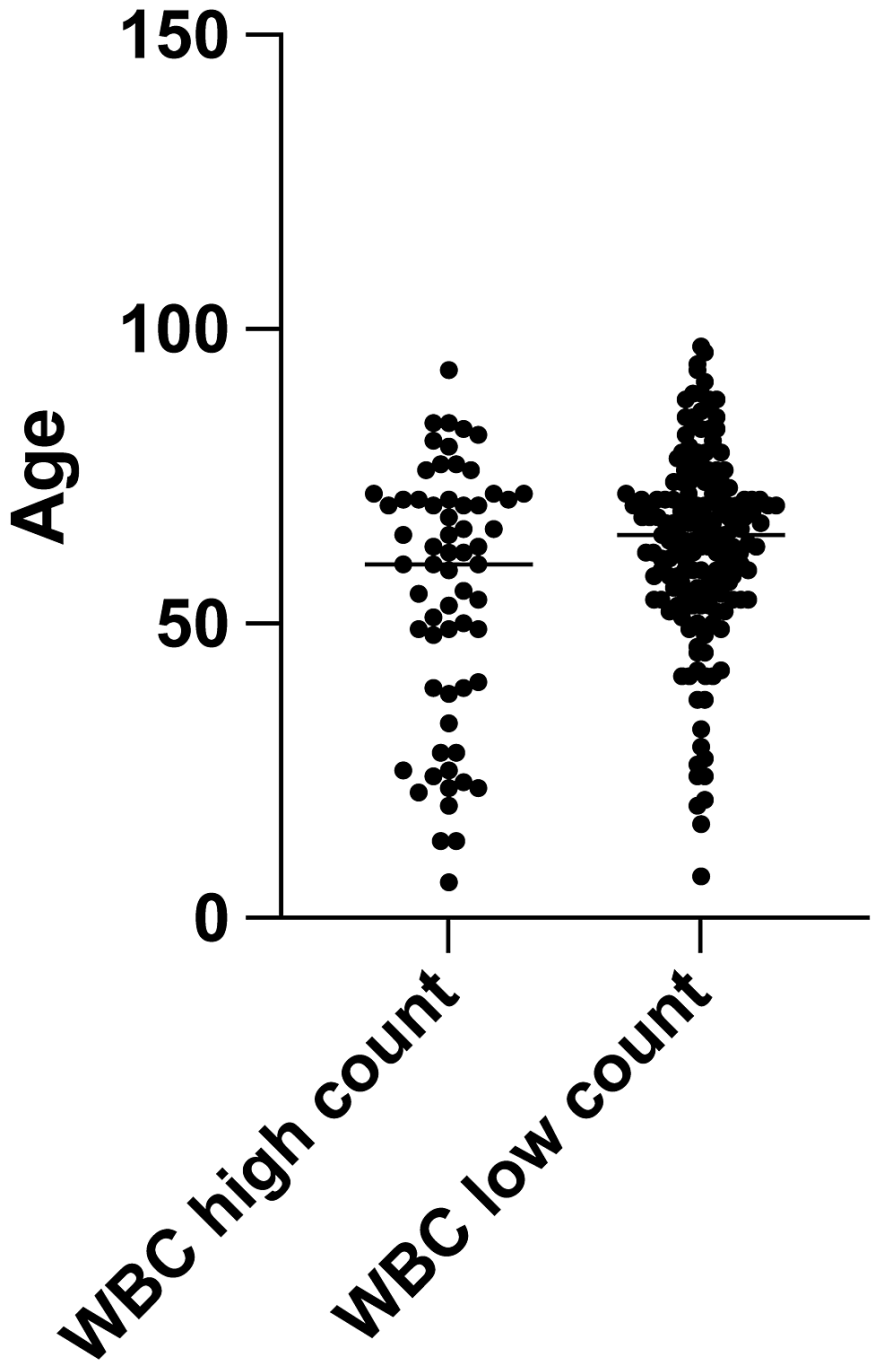
The relationship between age and high (>10,000 cells/uL) and low (<10,000 cells/uL) white blood cell (WBC) counts (unpaired t-test)

**Figure 4:**
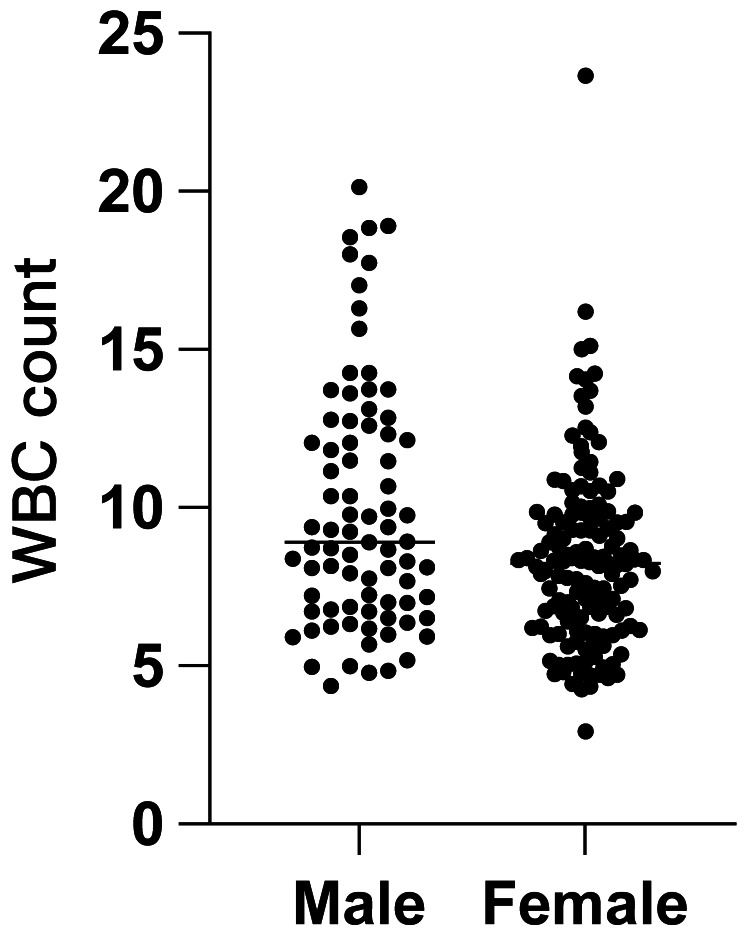
The relationship between sex and the white blood cell (WBC) count

We included 224 patients in this study (Table 1); there were more female patients than male patients. The WBC count did not differ based on the distal radius fracture location (i.e., the left or right side) (Figure 1; P > 0.05). Patients aged younger than 50 years correlated with an elevated WBC count (R2 = 0.04691; P <0.05) (Figure 2). However, the WBC count was not easily elevated in younger patients (cut-off values: WBC: 10,000 cells/uL, age: 50 years; P < 0.05, unpaired t-test) (Figure 3). The WBC count did not differ between males and females (P > 0.05, unpaired t-test) (Figure 4). Patients with Colles’ fractures showed elevated neutrophil and lymphocyte ratio (NLR) and WBC, but the patients with higher WBC showed dramatic elevation of NLR (For the patients with WBC < 10,000 cells/uL, the average NLR is 3.43; for the patients with WBC > 10,000 cells/uL, the average NLR is 6.74. P < 0.001, unpaired t-test).

**Table 1:** Baseline characteristics of patients with bone fractures

Characteristics	Open	
	n	%
Total patients	224	
Age, years (mean ± standard deviation)	61.37 ± 17.69	
Male	80	35.71
Female	144	64.29
Right radius, lower-end fracture	93	41.52
Left radius, lower-end fracture	102	45.54
Right radius, Colles’ fracture	13	5.80
Left radius, Colles’ fracture	16	7.14

## Discussion

We found that the incidence of distal radius fractures among women increased with age but remained low in men until they were older [9]. Therefore, surgical treatment might be more effective on the health and well-being of younger, non-elderly patients [10].

In the emergency room, patients with bone fractures often have an elevated WBC level. Therefore, it is challenging for emergency physicians to determine if the patient requires further testing for infection or if leukocytosis is due to pain and pressure after cast immobilization [11]. This study excluded patients with immune insufficiency, which could have affected the blood testing results. Elderly patients often have underlying diseases, including hypertension, type 2 diabetes mellitus, and chronic kidney disease, which affect liver and kidney function, thus the blood test results [12]. However, these conditions should not affect the WBC count. Leukocytosis is a common sign of infection, particularly bacterial infection, and should prompt physicians to search for other signs and symptoms [6]. Emergency physicians regularly check vital signs and blood test results before admitting patients to the hospital. Nonetheless, analgesic agents are often administered, and generally, a patient’s body temperature will be within the normal range, even if they fell due to weakness from infection. Our study found that pain and pressure after cast immobilization may induce a high WBC count in some patients. Also, the NLR is elevated while WBC elevated. That means WBC elevated because of high stress. NLR increases rapidly following acute physiologic stress (< 6 hours) [13]

All patients in our study were discharged. Deep infection after distal radius open reduction and internal fixation (ORIF) is less than 1% [14]. Santoshi et al. present the case of a patient with open fractures of the forearm bones due to horse bite. The open wound and animal bite increased the risk of infection. In this case, antibiotic prophylaxis played a key role to the successful management of the patient [15]. In our study, we exclude patients with open bone fractures. Thus, antibiotics may not be necessary for simple distal radius fractures treated in the emergency room.

This study has several limitations. First, fewer patients were under age 50 years than over age 50 years, likely because osteoporosis is more common in elderly patients. Furthermore, the study included fewer men than women. Hence, we can only describe a trend regarding the WBC count, NLR, and sex. 

## Conclusions

From review of medical charts, chest X-ray was done before admission (it is orthopedic ward pre-admission processes in Taiwan). No obvious infection sign was found via chest x-ray. Blood cultures of some patients were also collected if patients revealed fever and leukocytosis at the same time after admission. However, none of these patients showed infection, which was based on blood culture result. All these patients in our study were discharged.

Simple distal radius fractures are commonly treated in the emergency room, and some patients have elevated WBC counts owing to the pain and pressure from cast splint immobilization. Therefore, antibiotics may not be necessary for patients with simple fractures.
